# 30 A Retrospective Review of Outpatient Pain Management in the Burn Injured Population

**DOI:** 10.1093/jbcr/irac012.033

**Published:** 2022-03-23

**Authors:** Kevin N Foster, Philomene Spadafore, Anthony Mongelluzzo, Karen Richey

**Affiliations:** The Arizona Burn Center Valleywise Health, Phoenix, Arizona; Arizona Burn Center Valleywise Health, Phoenix, Arizona; Arizona Burn Center Valleywise Health, Phoenix, Arizona; Arizona Burn Center Valleywise Health, Phoenix, Arizona

## Abstract

**Introduction:**

Burn injuries and their associated treatments are exquisitely painful and often require hospitalization to achieve adequate pain control. Hospitalized patients have their pain managed aggressively, with rigorous monitoring. However, once patients are discharged from the hospital, the assessment of ongoing pain management becomes more difficult. The purpose of this study was to examine our current practices in the outpatient setting regarding pain management.

**Methods:**

This was a retrospective review of patients treated in the outpatient burn clinic following discharge home from the burn center over a one-year period. Patients with a significant history of premorbid pain and those discharged initially to a post-acute facility were excluded. Patients were stratified into those requiring opioids ≤ 4 weeks/ > 4 weeks, autografting/no autografting, opioids required following 95% wound closure/not required and those with and without a history of significant substance abuse.

**Results:**

There were 206 evaluable patients in this study. The mean number of days to healing was 19.5 days. The mean percent total body surface area injured was 5% yielding a mean number of days to healing per percent total body surface area burn of 4. Overall, narcotic pain medications were discontinued 8 days prior to wound closure. The majority of patients, 83% (n=170) had their narcotic pain medications discontinued before 95% wound closure was achieved; 17% (n=36) number of patients had their narcotic pain medications continued beyond wound healing. Subgroup analysis revealed the following findings. Patients who were on narcotic pain medication > four weeks following the time of injury (n=37) had a mean duration of narcotic pain therapy of 57 days compared to 11 days in patients who received ≤ four weeks (n=169, p < .00001). Likewise, patients who were grafted had a longer narcotic pain duration, 28 days vs 12 days in patients who were not grafted (p < .00001). Finally, the presence of previous or current substance abuse appeared to have no impact on the duration of narcotic therapy 19. 8 days vs 19.3 days.

**Conclusions:**

Most patients followed in the burn outpatient clinic demonstrated cessation of narcotic analgesic use around the same time as burn wound closure. Factors tending to extend the duration of narcotic use included longer inpatient narcotic use, and the necessity of skin grafting for burn wound management. Interestingly, previous substance abuse history did not influence length of post-hospital narcotic use.

